# Additively Manufactured Hierarchical Auxetic Mechanical Metamaterials

**DOI:** 10.3390/ma15165600

**Published:** 2022-08-15

**Authors:** Ekaterina Mazur, Igor Shishkovsky

**Affiliations:** Skolkovo Institute of Science and Technology, 121205 Moscow, Russia

**Keywords:** auxetic materials, metamaterials, additive manufacturing, energy absorption, meta-implants

## Abstract

Due to the ability to create structures with complex geometry at micro- and nanoscales, modern additive technologies make it possible to produce artificial materials (metamaterials) with properties different from those of conventional materials found in nature. One of the classes with special properties is auxetic materials—materials with a negative Poisson’s ratio. In the review, we collect research results on the properties of auxetics, based on analytical, experimental and numerical methods. Special attention of this review is paid to the consideration of the results obtained in studies of hierarchical auxetic materials. The wide interest in the hierarchical subclass of auxetics is explained by the additional advantages of structures, such as more flexible adjustment of the desired mechanical characteristics (the porosity, stiffness, specific energy absorption, degree of material release, etc.). Possibilities of biomedical applications of hierarchical auxetic materials, such as coronary stents, filtration and drug delivery systems, implants and many others, where the ability for high-precision tuning is required, are underlined.

## 1. Introduction

Modern additive manufacturing technologies allow for the creation of complex geometry at micro- and nanoscales. This ability makes it possible to produce new materials with properties different from those of conventional materials found in nature. Currently, many new metamaterials [1,2,3,4] are being developed to be used for specific engineering applications. In this paper, one of the classes of metamaterials, called auxetics, is considered. Unlike conventional materials, auxetic materials have a negative Poisson’s ratio, meaning that the material contracts in transverse directions under uniaxial compressive loads.

First proposed by Lakes [5], auxetic materials were intensively reviewed in a number of studies [6,7,8,9]. It was shown that auxetic material behaves differently to ordinary materials found in nature with a positive Poisson’s ratio under compression along the longitudinal direction: auxetics are characteristic of shrinkage instead of expansion [10]. The other high-performance mechanical properties of auxetic metamaterials, such as shear stiffness [11], fracture toughness [12], energy absorption [13], acoustic absorption [14] and compressive strength [15], were discovered.

The high-performance mechanical properties make auxetic materials desired candidates for many industrial and engineering applications, such as biomedical devices and implants [16], sensors [17], tissue engineering [18], blast protection [19] and many other applications. In this review, we consider the research results on the properties of auxetics, based on analytical, experimental and numerical methods. 

The general mechanism of auxetic behavior includes bending of the struts and rotation of the nodes, while the properties of the result material vary depending on the geometrical parameters that define the form of their elements. This review considered straight strut materials (particular cases of smooth geometry utilizing arcs, sinusoidal [20], Bezie curves [21], etc.) as they are sufficient to analyze the mechanism and base properties of the material. Several reviews on auxetic mechanical materials [22,23,24,25] have already been performed; however, the novelty of this review is in the consideration of the results obtained in studies of hierarchical auxetic materials. The wide interest in hierarchical auxetic materials can be explained by the additional advantages of structures, such as more flexible adjustment of the desired mechanical characteristics. 

This ability is highly desired in applications where a fine set of parameters is required. For example, for some implants, the detailed structures should mimic a natural organ or a bone in order to prevent stress-shielding problems. Special attention is paid to possible applications and bio applications of hierarchical auxetic materials, such as coronary stents, filtration and subsequent delivery systems, implants and many others, where the ability for high-precision tuning is required due to the human sensitivity level.

This paper starts with a section dedicated to a review of recent results in the research of auxetic metamaterials. Further, hierarchical auxetic structures are defined, and their properties and benefits found in the literature are discussed. We consider the advantages of several hierarchical structures: honeycomb hierarchical, re-entrant hierarchical, re-entrant honeycomb hierarchical, strengthened re-entrant auxetic materials and auxetic materials based on a rotating unit mechanism. 

The next section includes the most promising applications of auxetic metamaterials (both hierarchical and non-hierarchical). In conclusion, there is a summary of known auxetic and hierarchical auxetic properties and applications and further areas of structures investigation.

## 2. Auxetic Metamaterials

In many studies, the aim of the work is either to create a model of a new material that is compliant with experimental data or to improve the mechanical properties of an already existing auxetic metamaterial.

**Figure 1:**
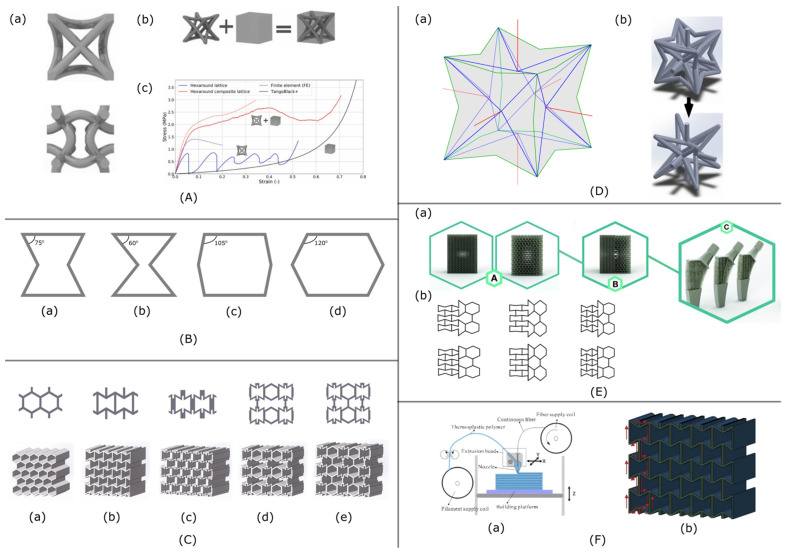
Auxetic structures. (**A**): (**a**) Hexaround (top) and Warmuth (bottom) cells in 2D. (**b**) Filling strategy. (**c**) Stress–strain graph for Hexaround lattice, filler and composite [26]. (**B**): Schematic design of individual cell dimensions for: (**a**) −15° re-entrant cells, (**b**) −30° re-entrant cells, (**c**) +15° honeycomb cells and (**d**) +30° honeycomb cells. (**C**): (**a**) honeycomb, (**b**) re-entrant and (**c**–**e**) hybrid honeycomb structures [15]. (**D**): (**a**) An idealized 3D re-entrant unit cell with its struts colored in red, blue and green depending on the strut type, (**b**) general and lightened re-entrant structure cells [27]. (**E**): (**a**) Schematic drawing of (A) auxetic and conventional meta-biomaterials, (B) hybrid meta-biomaterials and (C) meta-implants; and (**b**) design of six hybrid meta-biomaterials [28]. (**F**): (**a**) FDM printing setup and (**b**) schematic of CFRTPC auxetic honeycomb with specific printing path marked in red lines [29].

Auxetic composite lattices created by the addition of elastomeric matrix to the auxetic lattice of Hexaround and Warmuth structure (Figure 1(Aa)) were studied by Frederic Albertini et al. [26] who demonstrated experimentally and numerically that not only the increase in the Young’s modulus and Poisson ratio is gained but also higher plateau and peak stress compared to these parameters for lattice and filler separately. 

We found that composite structures (Figure 1(Ab)), printed using Material Jetting Technology (MJT) and constructed from lattice structure skeleton made of VeroWhite material (composition: isobornyl acrylate, acrylate monomers, urethane acrylate, epoxy acrylate, acrylate oligomers and photo-initiators) and compliant filler made of TangoBlack+ material (urethane acrylate oligomers, exo-1,7,7-trimethylbicyclo [2.2.1] hept-2-yl acrylate, methacrylate oligomers, polyurethane resin and photo-initiators), have higher specific energy absorption and energy efficiency (Figure 1(Ac)).

Energy absorption is a specific property of auxetic structures. Richard Critchley et al. [30] examined it for re-entrant and honeycomb structures (Figure 1B) made from composite polymeric material thermoplastic polyurethane TPU-95A. It was shown that auxetic material can be used as protection from the blast wave. For the considered structures, it was shown that both honeycomb and re-entrant structures decrease the impulse response: 23% blast mitigation for–15 degrees auxetic material and 12%-for–30 degrees auxetic material. 

In addition, it was shown that only re-entrant structures offer a reduction in the peak transmitted pressure. Thus, as the blast damage depends both on the impulse and pressure, re-entrant auxetics can be effectively used for the reduction of blast consequences. However, it should be noted that the critical angle for impulse response was not identified by the authors, and the peak transmitted pressure requires further investigation.

The other way to increase energy absorption performance is to combine auxetic and hybrid honeycomb structures [15] (Figure 1C). It was shown that the compressive strength of the structure created with the combined approach (Figure 1(Cc)) that utilizes an auxetic re-entrant structure with thickened struts between the cells was 300% higher than the strength of the honeycomb design and 65% higher than the strength for re-entrant auxetic structure manufactured from the same ABS polymer material. The Poisson’s ratio of the new structure was lower than that for parent honeycomb (Figure 1(Ca)) and the re-entrant auxetic (Figure 1(Cb)) structures; thus, the part can absorb more energy compared with them.

**Figure 2:**
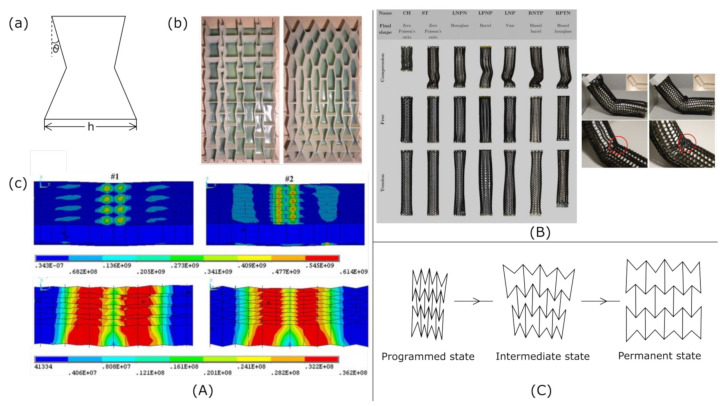
(**A**): (**a**) Topology of re-entrant unit cell with gradient geometry parameters: angle and horizontal length, (**b**) two geometric configurations of gradient honeycombs: #1 angle based gradient, #2 horizontal length based gradient and (**c**) nodal contour plots of Von Mises Stress of gradient sandwich panels [31], (**B**): Left: Deformation of the seven designs under tensional and compressive loads; right: Arm model covered with RNP design when (**a**) auxetic part and (**b**) hexagonal part are covering the internal part of the elbow [32]; and (**C**): Shape transformation assembled from reentrant honeycomb.

Not only energy absorption but also failure localization can be achieved with auxetic lattice. It was shown [31] that angle-gradient structures (Figure 2A) tend to localize the damage around the loaded area. Moreover, comparing different cell-wall aspect ratios and various internal angle gradient configurations, it was shown that the mechanical response could be tuned globally and locally by varying the geometry and the materials used for part fabrication.

The other way to study energy absorption effects is to consider ballistic performance. This property is crucial, for example, for the applications related to the process of bullets or projectiles caught. Hany Hassanin et al. [33] considered 3D re-entrant auxetic with superelasticity or shape memory capabilities (NiTi) in three stages of the impact process: top plate, auxetic structure and bottom plate penetration. It was shown that auxetic structure influences the dynamic response by becoming denser in the impact area, which increases energy absorption per unit mass at least two-times higher than that of a solid plate. 

Re-entrant 2D design can be extended to the 3D structure, which is supposed to be manufactured using additive manufacturing technologies. However, printing parameters (layer thickness, raster angle, etc.) and printing regimes influence the material mechanical characteristics [34,35]. Properties of 3D re-entrant structure are described with an analytical, based on Castigliano’s theorem model [36] that characterizes a prismatic auxetic material with a rectangular cross-section in which the material is subjected to uniaxial tensile load and defines Poisson’s ratio, elastic modulus and density ratio for the model material. 

The analytical solution complies with numerical simulation performed with finite element analysis in Abaqus software, which shows that stiffness properties, such as Poisson’s ratio and fracture toughness can be adjusted by cellular structure’s design parameters, and overall mechanical properties can be manipulated by the changes of the base wall angle of the configuration. Idealized 3D re-entrant structure [27] constructed of three strut types (Figure 1(Da)): one loaded purely axially and two loaded both flexural and axially, was also studied analytically, numerically, and experimentally. 

The authors introduced lightened re-entrant structure that at the same relative densities could be a better option due to its higher mechanical stiffness and strength. Additionally, it was shown that the lightened structure (Figure 1(Db)) could provide higher auxetic. A wide range of mechanical properties, such as Poisson’s ratio, elastic modulus and other mechanical characteristics make it possible to use the structures for implants with more compatible stress distributions. The special feature of the idealized structure is that there is no strut shared with adjacent cells; thus, it can be utilized in graded Poisson’s ratio distribution.

The other reason for the high interest and development of auxetic materials is their usage for biomedical applications. The comparison of implants made from conventional material, auxetic material and a combination of the two was performed by Helena M.A. Kolken et al. [28] who introduced hybrid material with a rationally distributed Poisson’s ratio that allows not retracting from the bone under biomechanical loading as occurs for conventional material. 

In general, hybrid material is obtained by joining two homogeneous auxetic and non-auxetic lattice structures and has an explicit or implicit boundary between parts with different Poisson’s ratios (Figure 1E). The experimental and simulation data have shown that under biomechanical loading, hybrid meta-implants press onto the bone on both the medial and lateral sides, and this way they improve implant-bone contact and influence implant longevity.

Specific research around auxetic structures in femur implants was conducted [37] as auxetics are helpful in dealing with the two most popular happening at the bone–implant interface: stress-shielding problem and the most important for implant longevity [38] micromotion problem, which is defined as the minimization of the relative motion of the implant with respect to the bone in its vicinity [39]. It was shown that auxetic implants with graded Poisson’s ratio distribution provide an opportunity to solve the mismatch of mechanical properties: stress and micromotion distributions between bone and implant.

The ability to expand in response to axial tension allows solving the problem of implant loosening. In another work, H M A Kolken et al. considered how auxetic meta-biomaterials [40] made from commercially pure titanium behave in cyclically loaded conditions, as the longevity of the implants directly depends on their properties under the conditions. 

Through micro-computed tomography, scanning electron microscopy and mechanical testing auxetic materials demonstrated morphological and mechanical properties appropriate for bone implants: elastic modulus range from 66.3 to 5648 Mpa, yield strength from 1.4 to 46.7 MPa, pore size range from 1.3 to 2.7 mm. Moreover, auxetic structures were superior to other non-auxetic structures made from the same material demonstrating an average maximum stress level of 0.47 σ_y_ at 10^6^ cycles of loading. These results prove the feasibility of using auxetic metamaterials for load-bearing implants, such as femoral implants, which currently need to be replaced over time in most cases due to changes in their mechanical properties.

3D-printed metallic auxetic material under cyclical load was studied by V.A. Lvov et al. [41] using finite element analysis and experimental static and low-cycle compression tests. Samples fabricated using selective laser melting technology from AlSi_11_CuMn powdered aluminum alloy have shown their ability to longer withstand cyclic loads than non-auxetic cellular samples. Auxetic material demonstrated uniform deformation in cyclic tests with a maximum load of 12 kN at a failure, compared with 8 kN for non-auxetic structure.

Cyclical loading of auxetic materials was also studied by V.A. Lvov et al. in [42]. Re-entrant samples from thermoplastic polyurethane were examined in low-cycle compression tests and demonstrated that the structure withstands 1.75 times more cycles than non-auxetic structure and there remain appropriate condition: no failure and no layer delamination were found in the samples after 500 cycles of compression. 

This behavior makes auxetic promising material for medical and sports applications [43] discussed in a separate section of this paper. Special attention was paid to the tubes manufactured from 2D auxetic material. In the research by Reza Hedayati [32], seven approaches of hexagonal and re-entrant structures were created for tubular specimens aiming at the creation of preprogrammed shapes upon application of external loads. It was shown that initially, the cylindrical tube changes its shape (Figure 2B left part) to symmetrical (vase, barrel, hourglass) and nonsymmetrical shapes (vase, hourglass) and that the experimental values of Poisson’s ratio are in good accordance with the analytical data [44]. This research proved the possibility to use auxetic tubes for distant actuation but also discovered new auxetic applications: wrinkle-free jointless hinges (Figure 2B right part).

Considering the creation of new material with non-conventional properties it is extremely important to take not only micro- but also nanoscale auxetic material into account [45,46,47,48]. Ultrathin 2D auxetic with geometry features of nano-scale size was fabricated [49]. This system was studied under mechanical loading conditions and showed in-plane dominated deformation up to 5% tensile strain and a Poisson’s ratio of −0.78, while porosity and aperture shape has changed significantly: pore sizes increased 4 times. This nano-scale behavior makes the system a candidate for nano-filters, such as a mechanically-induced tunable nano-filter for small particulate matter or microbes; however, the authors have not defined any particular filter design.

In order to improve the mechanical properties of auxetic material, Chao Quan et al. [29] have added fiber reinforced composite material into the structure. So-called continuous fiber reinforced thermoplastic composite (CFRTPC) auxetic honeycomb structures were fabricated in the FDM 3D printing process with two filaments extruded from one head (Figure 1F): thermoplastic polymer (PLA) and continuous fiber (Kevlar R). It was shown that the addition of a fiber increased the mass only by 6% but led to an increase in compressive stiffness and energy absorption by 86.3% and 100% respectively and smaller Poisson ratios.

Auxetic materials were also studied as materials for complex active structures that do not require assembly [50]. It was shown that auxetic materials fabricated from VeroWhitePlus RGD835 (VW+) are capable of achieving area changes up to 200%. With the thermoviscoelastic material complex active structures can be programmed into versatile shapes and recover their original shape given an external stimulus (Figure 2C).

In summary, auxetic metamaterials are of interest to researchers primarily because of their energy absorption properties. Most popular auxetic structures, such as honeycombs and re-entrants, show their ability to reduce impulse response in blast wave experiments, while re-entrants additionally reduce peak transmitted pressure. Moreover, composite materials consisting of auxetic skeleton structure and filler exceed the properties of the components separately providing a way to increase the energy absorption performance of the material. 

However, this is not the only way. As shown earlier, the combination of an auxetic re-entrant structure and a honeycomb structure in a certain way can produce a structure with a lower Poison’s ratio, which results in an increase in energy absorption performance. The other way is the addition of continuous fiber into the auxetic structure, which provides an increase in compressive stiffness and energy absorption as well. In addition, investigation of ballistic performance of auxetic with shape memory capabilities has shown the ability of the material to increase energy absorption per unit of mass due to the dynamic response of becoming denser in the area of impact. 

Furthermore, angle-gradient auxetics provide the ability to localize failure in the area of impact that can be used as an additional feature in a number of civil applications. Gradient auxetic materials or materials with a rationally distributed Poisson’s ratio are also of great interest because of their biomedical implant application where gradient auxetic metamaterial helps to resolve a number of problems happening with a conventional implant, such as implant–bone contact, stress shielding, micromotion and loosening under cyclical load, thus, increasing the longevity of the implants. 

The other promising property is shape transformation under the certain conditions that allow pre-programing of the shape that is reached upon the application of external conditions: load, thermal energy, etc.

## 3. Hierarchical Auxetic Materials

Hierarchical materials can be defined as a class of systems, which are composed of structural elements, which themselves have structure. Hierarchical materials demonstrate enhanced mechanical characteristics [51], such as lightweight high-strength characteristics and an increased resistance to crack propagation [52]. These properties are already used and highly desired in many bio-structures [53,54,55] and industrial and engineering applications [56,57,58].

**Figure 3:**
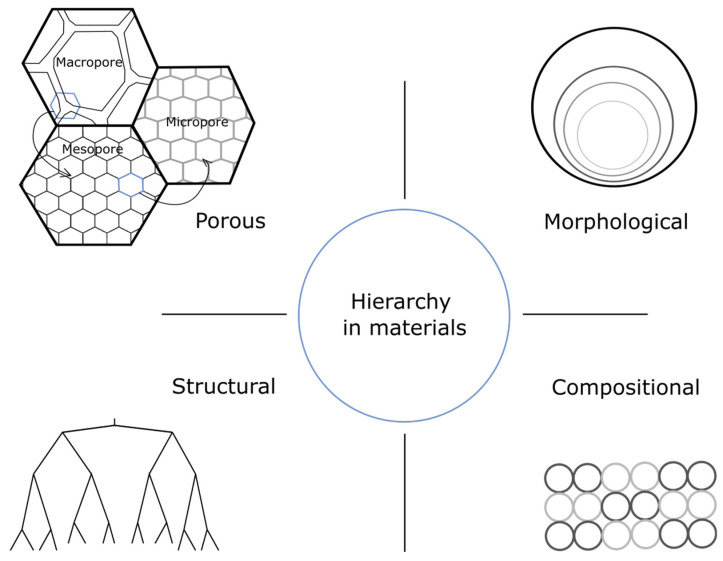
Types of hierarchy in materials.

In general, there are four main types of material hierarchy: porous utilizing multimodal porous structures with pore sizes at different scales, morphological created with multi-level microstructure units with specific morphology, structural including a strict and precise combination of structural elements and compositional utilizing assembly of small units (Figure 3) [59].

**Figure 4:**
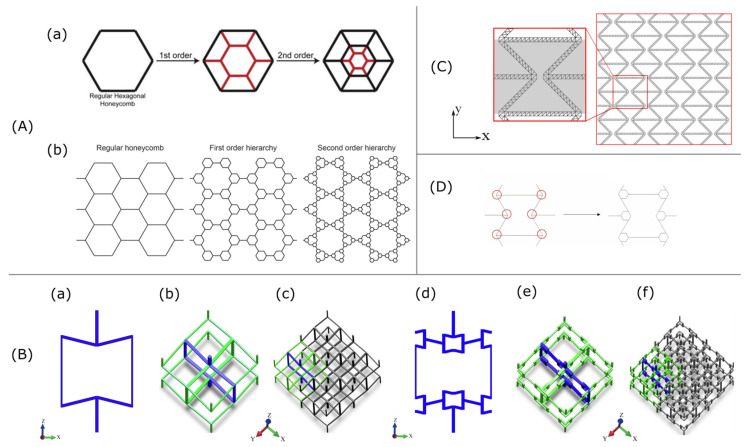
Hierarchical structures. (**A**): (**a**) Spiderweb structure [60] and (**b**) schematic showing the evolution of a regular hexagonal honeycomb and its corresponding cell into first and second orders of hierarchy [61]. (**B**): The schematics of re-entrant and hierarchical honeycomb structures: (**a**,**d**) unit cell in 2D, (**b**,**e**) unit cell in 3D and (**c**,**f**) the single-layer consisting of four cells [64]. (**C**): Globally auxetic lattice with minimally rigid sub-lattice [65]. (**D**): Modification of a conventional re-entrant unit cell (zero-order) to a first-order hierarchical unit cell [66].

However, there is inconsistency in the definition of the term hierarchy in the area of auxetic material research. Different authors define the hierarchy in auxetic structures in different ways. The hierarchy in the material structure itself means that the element or cell of one type of design is inclined in larger cells with the same or other design. Thus, different levels of hierarchy can be considered. As an example, hierarchical structures can be created from a cellular honeycomb lattice structure by substitution of the joints or nodes with cells of the same honeycomb design (Figure 4A).

There are two main ways to utilize the term hierarchy in auxetic structures. The first way assumes that the cells of each level of the hierarchy have auxetic properties. The second meaning allows having non-auxetic cells; however, the whole structure should have auxetic properties, and the resulting material should have a negative Poisson’s ratio under some strain conditions. In this review, the term hierarchy is not divided into two meanings; thus, all structures that satisfy at least one of the definitions were considered.

There are several main types of structures used for the creation of hierarchical auxetic material known and examined in the literature. Further, the main results are reviewed for each type of hierarchical auxetic metamaterial.

### 3.1. Honeycomb Hierarchical Auxetic

Mousanezhad et al. demonstrated [60] that “spiderweb” honeycomb structures that are a modified regular hexagonal honeycomb lattice material demonstrate auxetic properties at large deformations. Elements of the structure were created from a regular hexagonal lattice by adding smaller hexagons at the centers of cells of the underlying structure and connecting adjacent vertices with inner struts (Figure 4(Aa)). The authors introduced [61] 2D hierarchical honeycomb auxetic metamaterials of the structure shown in Figure 4(Ab) and show the role of hierarchy in the structure of auxetic materials based on experimental and computational data. Auxetic behavior was observed in the first order hierarchy, while it was absent in the non-hierarchical counterpart. 

Moreover, two deformation modes were introduced: X-shape, when the deformation is governed by elastic buckling of the horizontal cell walls and rotation of the corresponding smaller hexagons in the central hexagonal cell (similar to buckling of a hexagonal honeycomb in biaxial compression), and N-shape, when the zigzag collapse of hexagonal cells happens due to compression along the x-direction, similar to the uniaxial buckling mode of the regular hexagonal honeycomb [62,63]. Mousanezhad et al.’s analysis has shown that the materials with the first order hierarchy, which corresponds to a point in which the buckling modes switch, have the lowest Poisson’s ratio and can be decreased by the addition of higher orders of hierarchy.

### 3.2. Re-Entrant Hierarchical Structure

The theoretical model of re-entrant hierarchical metamaterial verified numerically and experimentally was proposed by Lumin Shen et al. [64] who developed a hierarchical structure, which can be created from regular re-entrant structure by the substitution of each vertex in the structure with a smaller re-entrant cell with self-similar configuration. (Figure 4B). It was shown that hierarchical re-entrant structure can have higher Young’s modulus and more comprehensive auxetic effect flexibility compared to conventional re-entrant lattice material.

### 3.3. Re-Entrant Honeycomb Hierarchical Structure

The Hybrid Hierarchical Re-entrant Honeycomb (HHRH) structure [66] was proposed by Ahmed Haytham Tajalsir et al. This structure combines re-entrant and honeycomb structures and can be created by the substitution of the vertexes of the re-entrant structure with honeycombs cells of a smaller size (Figure 4D). It was shown that HHRH material provides up to an 85% reduction in peak stress and increases the auxeticity by up to 23% when compared to the regular re-entrant auxetic structure under high-velocity applications.

### 3.4. Strengthened Re-Entrant Metamaterial

D. Rayneau-Kirkhope [65] examined the role of hierarchy in the auxetic lattice (auxetic structure with inclined right-handed lattice in the struts shown in Figure 4C) with a methodology involving a coarse grain approach and computational methods and showed that, with hierarchy, one can create ultra-lightweight auxetic meta-materials of high strength and stiffness.

### 3.5. Rotating Unit Mechanism

**Figure 5:**
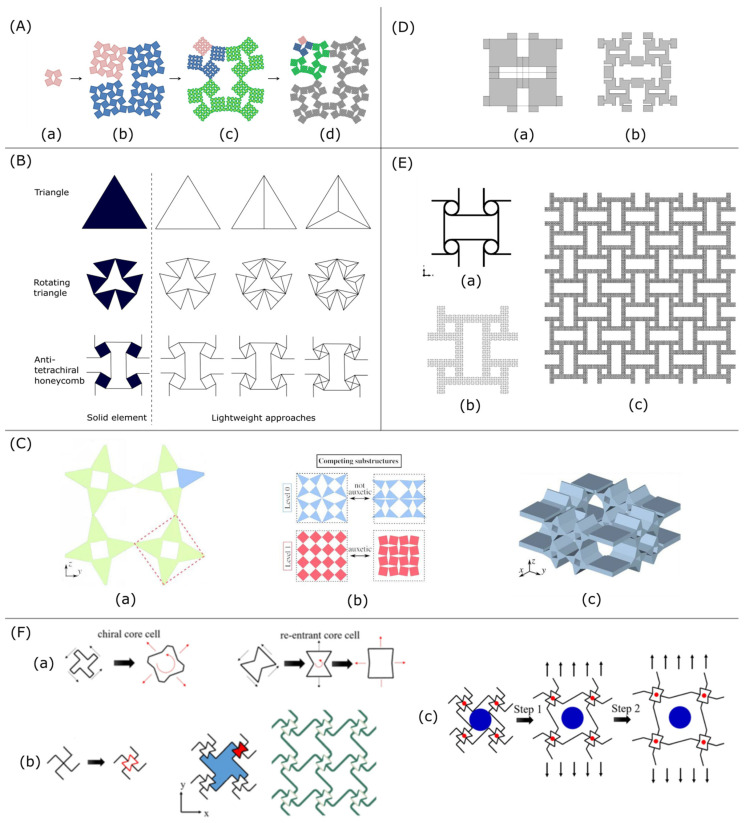
Utilization of rotating unit mechanism in material structure. (**A**): Diagram depicting hierarchical system based on the rotating rigid units mechanism: (**a**) one level system, (**b**) two level system, (**c**) three level system and (**d**) three level system part [67]. (**B**): Schematic design of possible approaches for substitution of rotating blocks with lattice cells for lightweight design of four auxetic rotating solid blocks structures. (**C**): The design of 2D and 3D hierarchical structure: (**a**) a cross-section of the unit-cell: in blue–level 0 element, in red–level 1 element, (**b**) conceptual deformation of substructures corresponding and (**c**) the unit-cell of the 3D model considered [68]. (**D**): (**a**) Geometry parameters of the base unit cell and (**b**) hierarchical, auxetic rectangular perforations 2 of the hierarchical structure [69]. (**E**): Model of second order hierarchical anti-tetrachiral metastructure: (**a**) anti-tetrachiral unit cell with circular ring node, (**b**) superior structure of anti-tetrachiral unit cell with square node and (**c**) material structure consisting of cells with square node [70]. (**F**): (**a**) The cell opening mechanism of a chiral core and re-entrant core, (**b**) the concept of generating a new chiral cell with re-entrant core cell (top) and the two types of cells (blue and red) in the new periodic chiral metamaterial (bottom) and (**c**) the sequential particle release mechanisms of red and blue dyes under external tension shown by arrows [71].

Ruben Gatt et al. [67] proposed a new type of hierarchical auxetic structure based on the rotating rigid units mechanism. The simple square multi-level rotating elements (Figure 5A) were considered, and it was shown that the auxeticity, degree of aperture and pores size can be controlled with the design.

The same mechanism was considered by Yichao Tang and Jie Yin [72], who studied thought modelling the geometry, experiments and simulation, so-called hierarchical “kirigami-based” auxetic structures produced with the introduction of line cuts (“kiri” means cut) into thin sheets of elastomer. A number of researchers [73,74] have shown that metamaterials manufactured this way perform highly stretchable and compressible material design, which exhibit highly nonlinear stress–strain behavior resulting from the hierarchically cut structure rather than constituent material. 

These properties are highly desired for the development of such applications as stretchable and conformable electronics [73] and stretchable energy storage devices, such as kirigami-based lithium-ion batteries [75]. These structures are based on rotating rigid mechanisms. It was shown that extreme stretchability via unit rotation increases in rectangle units (compared to the square unit) from 62% to 156% for the hierarchical structure, and moreover, in the experiment with the same design but with solid parts replaced with porous squares and re-entrant lattice shapes, a compressibility value of 81% was detected.

Lattice structures are naturally used for lightweight design. Luke Mizzi and Andrea Spaggiari [76] demonstrated that solid rotating blocks in non-hierarchical auxetic can be substituted by various lattice cells and proved (using experimental and numerical methods) that lightweight structures (Figure 5B) have almost no difference with full (solid) structures in the Poisson’s ratio.

#### 3.5.1. Hierarchical Rotating “Star” Metamaterial

The other property of hierarchical auxetic is shape morphing. Krzysztof K. Dudek et al. [68] demonstrated that auxetic behavior can be controlled by the structure design. Analyzing the experimental and simulation data for microscopic hierarchical metamaterials (Figure 5(Ca)), whose structure uses mutually-competing substructures on different levels of hierarchy (Figure 5(Cb)) within the system, showed that they were capable of exhibiting a broad range of highly unusual auxetic behavior. Additionally, it was demonstrated that the structure can be used as a morphing composite capable of deforming to a predicted shape.

#### 3.5.2. Perforated Chiral Auxetic Metamaterial

Similar to line cuts and cut-out approaches, mosaic perforation can be performed to create auxetic materials. Composite auxetics with the hierarchical structure created using the technique were examined [69] for wave propagation characteristics using the Floquet–Bloch method [77]. The authors proposed a perforation template to design a topology that is able to create a significantly broad range of full bandgaps and tailored center frequencies dependent on the hierarchy and geometry parameters of the cells (Figure 5D).

#### 3.5.3. Hierarchical Anti-Tetrachiral Material

The auxetic behavior of such a material is based on a chiral structure [78,79,80,81,82], where elements consist of circular ring nodes and tangentially connected ligaments. This structure is also used as a base for the other hierarchical auxetic structures—hierarchical anti-tetrachiral metastructures [70], each cell of which is made of four chiral cores with opposite rotating direction to the neighboring cores (Figure 5E). In the work of Wenwang Wu et al., it was shown that chiral hierarchical geometry (node ring size, shape, length and thickness) can be tuned to have predefined auxeticity and mechanical properties.

#### 3.5.4. Combination of Chiral and Re-Entrant Auxetic Material

The other hierarchical auxetic material that utilizes chiral structure was introduced by Yunyao Jiang and Yaning Li [71]. Their hybrid structure (Figure 5(Fb)) based on the re-entrant mechanism on the second level of hierarchy was additionally modified by the usage of multi-material printing. It was shown that the design can be used for subsequential cell-opening mechanisms (Figure 5(Fa)) under a large range of strains. This mechanism can be utilized in a number of applications: the sequential addition of chemicals in a closed system needed for desired reaction, color change, when dyes of various sizes are placed inside the cells and are sequentially released, staining the carrier in the desired color (Figure 5(Fc)), etc. Moreover, Poisson’s ration, stiffness and cell-opening rate can be tuned by two independent geometry parameters.

In summary, the hierarchical structures developed are based on the approach of the utilization of various cellular structures (auxetic and non-auxetic) in several but mostly two, levels of the hierarchy. This allows for the creation of a number of material designs with auxetic properties by inclining cells of one design into the units—for example, the vertices of the cellular structure of the same (honeycomb or re-entrant hierarchical material) or the other design (re-entrant honeycomb hierarchical material). 

In a number of studies, a rotating unit mechanism is used for one of the levels of hierarchy (chiral re-entrant hierarchical material, hierarchical anti-tetrachiral material, perforated chiral material, etc.). It should be noted that the usage of the rotating unit mechanism does not violate the approach defined above, because the rotating unit itself is a particular case of auxetic cell structure.

The usage of hierarchical structure provides additional benefits to the material considered. In this section, the observed benefits of different designs of hierarchical materials were considered. For example, for hexagonal honeycomb hierarchical material, it is the lowering of the Poisson’s ratio and the observation of two deformation modes that are different to non-auxetic honeycomb materials. For re-entrant hierarchical material, it is an increase in Young’s modulus value and observation of a comprehensive auxetic effect. 

Combined re-entrant and honeycomb structure in a certain way can deliver an increase in auxeticity and a reduction in peak stress. Moreover, a simple inclining of a lattice structure into re-entrant struts provides strengthening of the material and an increase in the stiffness values. The other advantages of hierarchical structures mainly utilize a rotating unit mechanism: high stretchability and compressibility, highly nonlinear stress–strain behavior, shape morphing capabilities, subsequential opening, etc.

## 4. Applications

Auxetic material is an appropriate candidate for bone implant material as it helps to solve all main problems that occur with implants manufactured from conventional material including stress concentration and micromotion issues. For example, an idealized re-entrant material [27] can be easily utilized for the creation of gradient Poisson’s ratio distribution. Thus, the auxetic metamaterial can be used in designing and manufacturing meta-implants, where such a graded ratio distribution helps to enhance the micromotion at the bone–implant interfaces.

Analysis of the usage of the auxetic structure made of thermoplastic polyurethane and examined under cyclical loading [42] has shown that it can be used in the development of structural elements of biomedical devices, such as spinal implants and bone implants for supporting limbs [43].

**Figure 6:**
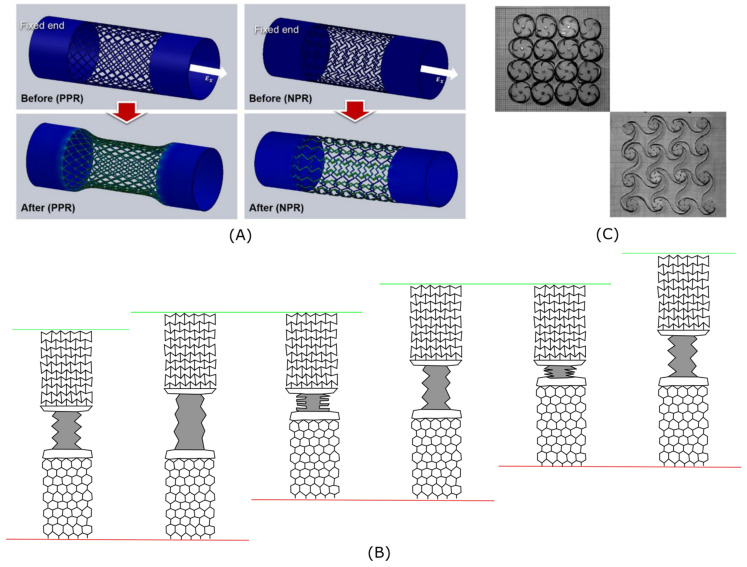
Applications. (**A**) Blood vessel with non-auxetic and auxetic material before (top) and after (bottom) the load [84]; (**B**) schematic representation of the locomotion of soft material robot: during the expansion the auxetic clutch moves upwards (green line), and the normal clutch is stationary (red), during contraction the auxetic clutch remains fixed, and the normal clutch slides upwards; and (**C**) folded and unfolded auxetic cellular antenna [84].

One of the most important biomedical applications of the metamaterial is blood vessels [83] and coronary stents, which are able to increase the wall thickness in response to a pulse of blood (Figure 6A). This ability prevents the rupture of the device and its further replacement.

### Coronary Stents: From Design to Mechanical Testing

**Figure 7:**
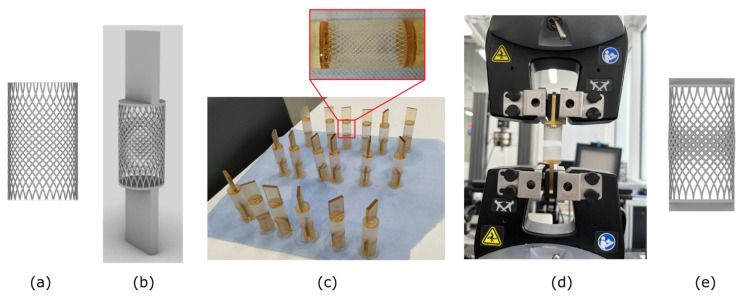
Manufacturing and testing of stent samples stages: (**a**) lattice design, (**b**) sample design, (**c**) printed parts: three samples for each of six modifications, (**d**) broken sample after mechanical tensile test and (**e**) proposed improved design with gradient lattice design.

Samples of the coronary stents that are widely used in cardiac surgery with an inner lattice structure were fabricated at the Skolkovo Institute of Science and Technology in Additive Manufacturing Lab at Center for Materials Technologies (Figure 7). Six different models were designed in Rhino 7 and Grasshopper varying two key parameters: the number of fibers and fiber radius. Inner structure management and/or radius gradient creation can bring to auxetic properties into such scaffolds. The stents were manufactured from Anycubic 3D printing UV-sensitive resin material using Zortax Inkspire 3D Printer installation (digital light processing (DLP) technology) and tested via an Instron 5269 testing machine.

Each set of samples included the following modifications: 0.25 and 0.30 mm were the radii of wires inside the coronaries and 24, 29 and 34 were the numbers of wires used in the proposed design. During the design process, several problems need to be considered. First, there is an overhang angle limitation because of the chosen printing technology. Second, there is a connection problem that occurs between the solid part and the lattice part of the sample. This is a weak place of the model where it might break potentially. In order to solve the problem lattice is sunk into a solid part so that the cross-section area of the lattice’s contact with the solid is increased.

Mechanical testing showed that the lattice design parameters significantly influence the mechanical behavior of the samples: the sample with a wire radius of 0.25 mm and wire number of 29 had the highest detected Young’s modulus: 383 MPa compared to 24.2 MPa—the minimum determined in the experiment (for the sample with wire radius 0.3 mm and a number of wires of 29). The design is a middle ground between a number of wires and wire radius in the selected set of parameters, as the addition of more wires and/or increasing wire radius makes the specimens less stiff.

During mechanical testing, the majority of samples broke in the middle area of the stent’s lattice (Figure 7d). In order to improve the mechanical properties (for example, to increase the limit load of the samples), advanced design could be required in future. One of the solutions is the usage of a gradient lattice. There are two possibilities for realization: additional wires can be added inside the lattice, or the wires radius can be increased in the middle of the sample (Figure 7e).

The other biomedical application is a special bandage [85] that differs from the conventional one in that it is not needed to be changed in order to apply medication. The subsequential mechanism allows the application of treatment based on the blood absorption or manipulation with external conditions (the press, tension, etc.). Hierarchical auxetic material based on rotating units [67] is more versatile compared to similar non-hierarchical, lattice materials; thus, this is a candidate for skin grafts and stent manufacture.

The mechanism of subsequential cell opening [71] has potential applications in drug delivery, where several separate stages of drug treatment are required sequentially. The presented hierarchical design concept can be used to develop new multi-functional smart composites, sensors and actuators responsive to external conditions. The hierarchical auxetic “kirigami-based” materials [72] mentioned above can be utilized for the creation of flexible, conformable and stretchable electronics, biomedical devices, electronic skin, reconfigurable soft robotics, etc.

Reviewing soft robotics applications, the ability of auxetic structures to reactively change in shape when moving through a constriction should be considered. This property can be a means of achieving compliance [86] that allows robots to overcome challenges by deforming and conforming their bodies. Thus, the use of auxetic materials reduces the complexity of control, allowing a robot to change shape autonomously, with potential applications in the exploration of unpredictable territory and subterranean or bounded spaces, such as the human body.

For example, an inchworm-type soft robot for crawling through channels exploiting mechanical metamaterials for the intrinsic synchronization of two passive clutches was presented in [87]. The design allows it to move through an enclosed passage with an inchworm motion propelled by a single actuator. The sequence of images (Figure 6B) shows three cycles of expansion and contraction of the bellows. During the expansion, the auxetic clutch moves upwards, and the normal clutch is stationary. During contraction, the auxetic clutch remains fixed, and the normal clutch slides upwards.

The passive clutch mechanism based on complementary material properties helps to reduce the number of actuators to one and to avoid synchronization problems as the smart synchronization is already embedded in the robot’s material body. Such a robot can be used for navigating within bores that are soft themselves, cylindrical tubes of unknown cross section and other related applications.

Substitution of the rotating rigid with lattice cells makes the material lightweight [76] and makes it an ideal candidate for implementation in applications requiring lightweight auxetic metamaterial systems, such as in the aerospace industry and robotics systems.

The other auxetic ability to be programmed [50] into versatile shapes and recover their original shape given an external stimulus may be further used for applications, such as biomedical devices, civil structures, and aerospace. For example, a chiral-based honeycomb deployable antenna [84] for deep-space missions (Figure 6C) utilizes shape memory properties. Initially, it is set in a limited space for transportation and rocket launch. However, as soon as there is enough space or it is already in space, the structure reconstructs itself to the original size utilizing heat energy or the thermal energy of the sun in the case of space.

External stimulus can be not only loading or thermal; however, it can also be magnetic, electrical, etc. For example, magneto-active properties of the materials occur due to magnetic micro or nanoparticles distributed within a soft polymer matrix, and when the magnetic field is applied, some torque is generated on magnetic soft materials until the magnetization direction of all domains is aligned with the field direction [88]. Thus, programmable shape deformation [89] can be created in the material via the spatial distribution of magnetic particles inside. Shape-morphing magnetic soft machines are desirable for minimally invasive medicine, wearable devices and soft robotics applications.

In recent years, artificial piezoelectric materials have been proposed, in which the piezoelectric response of materials was explained to their special geometry and/or structure [90,91]. In this case, since the piezoelectric response was not connected with the chemical composition but has been described to the topological structure and geometry, such material can be considered as auxetic metamaterials. There is information [90] that the piezoelectric response can demonstrate stability up to high temperatures in those piezoelectric metamaterials. The design of topological and geometric structures with extended and artificially defined parameters (negative, close to zero, ultra-high and/or customizable), i.e., extraordinary auxetic properties, is a great challenge in the field of metamaterials.

For natural piezoceramics, there are five non-zero elements in the dielectric constant matrix. Yang et al. [92] showed that, by using a quasi–symmetry violation and realizing artificial anisotropy during metamaterial design, it is possible to excite all non-zero elements. Carefully theoretically and experimentally modelling topological structures and geometric sizes of single elements, Yang et al. obtained customized non-zero or ultra-high values of general effective piezoelectric coefficients. 

The possibility of constructing several main types of anisotropic topological structures in the lattice form was shown in [93] as well as the ability to combine them jointly (creation of hybrid structures). Anisotropic individual and hybrid lattices (pentamode metamaterials) were made by the Multi Jet 3D printing and then were subjected to mechanical compression tests (Instron 5969). The FEM for such structures were developed via COMSOL Multiphysics. As a result, the possibility of designing and constructing extreme materials with arbitrary eigenvalues in the tensor of elasticity was shown. It was concluded that the elastic-E, shear-G and volume-B modules of such hybrid metastructures are a superposition of the corresponding modules of individual lattice structures. 

The Poisson coefficient of a designed hybrid pentamode metastructure is equal to the Poisson’s coefficient of an individual structure but with a higher Poisson ratio. The yield strength of the hybrid pentamode lattice structure was dependent on the elasticity modules of separated lattice structures, and the yield strength of the lattice structure had a lower order. Future applications of such piezoelectric metamaterials in MEMS applications include new types of actuators, ultrasound engines, sensors, and energy harvest generators. [91,94].

In general, shape morphing auxetics [68] can be used in applications where the material can significantly change its mechanical response—for example, in flexible electronics [95,96] and for specialized medical equipment, such as coronary stents [97] and implants as well [98].

As auxetic materials and especially composite auxetic materials [26] have higher specific energy absorption and energy efficiency compared to conventional materials they can be used for the creation of new energy absorption devices based on the material structure.

Energy absorption is also a desired property in civil engineering. Concrete, masonry and mortar used as building materials are brittle in nature and can be easily fractured by impact, blast and seismic forces. Auxetic materials, as high energy-absorbing materials, can strengthen the building structures, thus, reducing the damage and hence the loss of lives and repair costs. It was shown experimentally [99] that mortar-auxetic composites exhibited high energy absorption and high bonding strength with the mortar in comparison to the common fiber-reinforced polymer (FRP) composites used for strengthening the building materials. 

Moreover, simulation using the finite element modelling method confirmed that the brick masonry wall rendered with mortar-auxetic composites performed better in resisting the lateral impact in comparison to the un-rendered wall resulting in the lateral displacement reduction by 22%, and the energy absorption increased eight times. These results show the high potential of the utilization of auxetic materials in civil structures for strengthening and protection purposes.

Moreover, it was shown [61] that hierarchical auxetic material can be used not only for energy absorption devices but also for tunable membrane filters with variable permeability that allows to use geometry parameters of the filter material for the control of the passage pressure [100] and acoustic dampeners.

## 5. Conclusions

Auxetic structures are promising materials due to their special properties. In this article, significant results obtained in the research of auxetic materials were considered, and existing and possible applications were suggested based on the literature reviewed.

To summarize, one of the main advantages of auxetic metamaterials and especially hierarchical auxetic materials is that, with the help of the design of the material structure, and setting its geometric parameters, it is possible to influence and fine-tune its mechanical properties. This makes the materials desirable in many industrial and biomedical applications: industrial applications where exceptional fineness is needed and medical and biomedical applications where accuracy is needed due to human sensitivity or the customization of medical devices. Existing and possible applications were suggested based on the literature reviewed.

The other property that creates high demand for auxetic materials is increased energy absorption in comparison with conventional materials. The ability to decrease the impulse response, peak transmitted pressure, localize failure and absorb more energy per mass unit due to becoming denser in the impact area makes the materials potential candidates for utilization in applications related to protection and damage minimization.

Moreover, angle-gradient auxetic materials help to solve the main problems of conventional bone implants: implant-bone contact, stress shielding, micromotion, implant loosening and allow for the creation of long-term implants that do not need to be removed during the lifetime of the patients.

In addition, there is an approach for the prediction of shape transformation of auxetic material structures under certain conditions. This includes structures made of shape-memory materials or structures that change their shape under external loads. The prediction gives an opportunity to fabricate or transfer the structure in a limited space and use it in the predicted shape when needed.

Hierarchical structures described in the literature mostly utilize two levels of the structure by inclining cells of one design into the cellular structure of the same or the other design. At least one of the cells should have auxetic properties in order for the material to behave accordingly. The hierarchical level creates an additional advantage that can be controlled by the geometrical parameters of the structure. The main benefits include a decrease in the Poisson’s ratio, an increase in the energy absorption, the creation of an additional mode of material behavior, an increase in the Young’s modulus, increased stretchability and other improvements as discussed in the review.

All the experiments, simulations and analytics described in the literature deal with hierarchy orders less than or equal to two. An additional level in the hierarchy increases the flexibility in mechanical parameters tuning; however, it also increases the model complexity. Higher-order structures can be analyzed in the future.

## Data Availability

Not applicable.
